# A Case of Tachycardia-Induced Cardiomyopathy During Pregnancy: Clinical Presentation and Management

**DOI:** 10.7759/cureus.33229

**Published:** 2023-01-01

**Authors:** Andrii Labchuk, Emily Temple-Wood, Daniel Sherlock, James Russell, Marianna Krive

**Affiliations:** 1 Internal Medicine, Advocate Lutheran General Hospital, Park Ridge, USA; 2 Family Medicine, Advocate Lutheran General Hospital, Park Ridge, USA; 3 Cardiology, Advocate Lutheran General Hospital, Park Ridge, USA

**Keywords:** antiarrhythmics, tachycardia in pregnancy, tachycardia induced, pregnancy care, atrial tachycardia

## Abstract

A 28-year-old G2P0010 woman with a history of COVID infection during her current pregnancy treated with monoclonal antibodies and benign gestational thrombocytopenia presented for routine prenatal care at 33 weeks' gestation. The patient was asymptomatic, but incidental tachycardia was noted on the physical exam with an irregular rhythm. An electrocardiogram (ECG) was performed and was consistent with multifocal atrial tachycardia at a rate of 144 beats per minute. The patient was started on labetalol 50 mg daily and was referred to cardiology for consultation. An echocardiogram was performed and showed dilated left ventricular cavity with a moderately reduced ejection fraction of 40%. No previous echocardiogram was available for comparison; the patient had no history of cardiac disease. The dose of labetalol was increased to 50 mg twice daily and she was admitted for digoxin loading and titration. Though fetal tolerance was excellent, her heart rate was not controlled. Digoxin was switched to flecainide and labetalol was switched to metoprolol which improved her heart rate and repeat echocardiogram showed an ejection fraction of 50%. The patient was admitted for induction of labor at 39 weeks of gestation and continued intrapartum flecainide. Metoprolol was continued intra and postpartum. Flecainide was resumed at three days postpartum due to the recurrence of atrial tachycardia and has been maintained. A repeat echocardiogram is scheduled six weeks postpartum to evaluate left ventricular function and wean off antiarrhythmics.

## Introduction

During pregnancy, there is a physiologic increase in heart rate due to the increase in circulating volume and the necessity of higher cardiac output. There is no clear definition of “physiological tachycardia” during pregnancy which can lead to interpreting any elevated heart rate during pregnancy as a variant of normal. Long-standing tachycardia can lead to serious complications. Tachycardia-induced cardiomyopathy is a reversible dysfunction of the myocardium that occurs due to sustained elevation in heart rate. It is important to remember that developing symptoms of left ventricular failure also could be masked under symptoms of pregnancy like weight gain, shortness of breath, and peripheral edema. Monitoring heart rate during routine prenatal care can help providers screen patients who need further evaluation with electrocardiography, a cost-effective way to evaluate arrhythmias. If a tachyarrhythmia is diagnosed, an echocardiogram should be performed to evaluate for structural heart disease and ventricular dysfunction.

## Case presentation

A 27-year-old G2P0010 presented to the family medicine office for routine prenatal care after a positive home urine HCG test. Initial ultrasound showed a live intrauterine pregnancy at 11w2d by a crown-rump length with normal fetal nasal bone and normal nuchal translucency. Prenatal blood work revealed blood type O negative without alloantibodies and varicella zoster non-immunity. Other blood tests including rapid plasma reagin, complete blood count, hepatitis serologies, rubella antibodies, and Papanicolaou testing were unremarkable. Her pregnancy progressed without complication until 16w5d when she tested positive for COVID-19, for which she was treated with casirivimab-imdevimab monoclonal antibodies on an outpatient basis. The patient was vaccinated and received one booster for COVID prior to her pregnancy. She recovered well and noted no residual symptoms at follow-up prenatal care. An anatomy ultrasound conducted at 18w3d revealed an anatomically normal male fetus measuring an estimated 261 g (68th percentile). Maternal-fetal medicine recommended routine growth ultrasound at 30-32 weeks due to COVID infection. The glucose tolerance test conducted at 24 weeks gestation was normal. At 24 weeks, routine CBC was obtained, and mild thrombocytopenia was noted (platelet count 131,000), thought to be consistent with benign gestational thrombocytopenia. The patient was normotensive, without proteinuria, and had normal liver enzymes, which were not consistent with immune thrombocytopenia, pre-eclampsia, or HELLP syndrome. She was treated with prophylactic Rho(D) immune globulin at 26 weeks' gestation and routine CBCs were obtained at subsequent prenatal visits. Throughout the remainder of the pregnancy, her platelet counts remained at or above 99,000.

**Figure 1:**
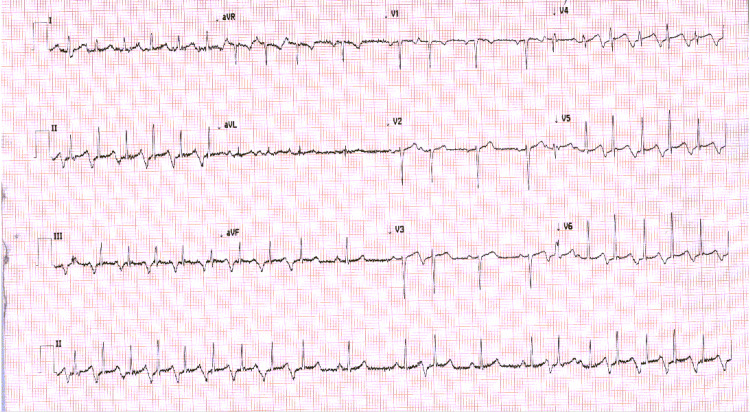
Initial ECG on presentation

At 32w3d the patient underwent a growth ultrasound which revealed a live fetus measuring 2074 g, 55th percentile for gestational age. Routine follow-up and prenatal care were recommended by maternal-fetal medicine. At 33w0d, she presented routine prenatal care without complaint, but she was noted to be tachycardic to the 140s on presentation. ECG was obtained and showed atrial tachycardia (Figure 1).

**Figure 2:**
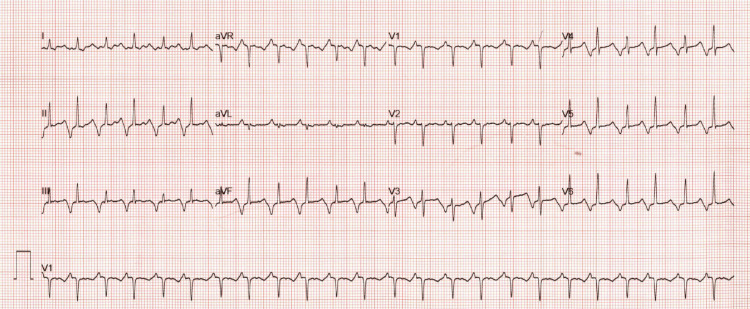
ECG obtained during the appointment with cardiologist

The patient was completely asymptomatic. She started on labetalol 50 mg PO daily and urgent cardiology consultation was obtained. At 33w4d, ECG again showed a paroxysmal atrial tachycardia with a rate of 144 BPM and frequent conversion to sinus rhythm (Figure 2). Echocardiogram was obtained and revealed left ventricular ejection fraction of 40%.

Same-day electrophysiology consultation was obtained, and the patient was admitted to our institution for continuous fetal and ECG monitoring during a digoxin load. During that admission she was continued on labetalol during digoxin loading which did not control her heart rate despite therapeutic trough levels.

**Figure 3:**
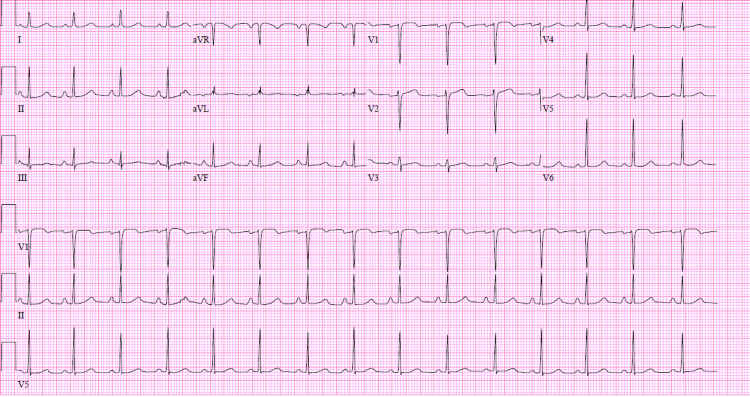
ECG after treatment with flecanaide

Continuous electronic fetal monitoring was normal for gestational age throughout the admission. The patient was started on flecainide 50 mg twice daily and metoprolol tartrate 25 mg every eight hours. The patient converted to sinus rhythm on hospital day four (Figure 3) and maintained normal sinus rhythm until her discharge on the same medication regimen with ambulatory telemetry that sinus rhythm with atrial tachycardia.

She returned to the cardiology, family medicine, and maternal-fetal medicine clinic for close follow-up and repeat echocardiogram was obtained at 36 weeks' gestation showing a left ventricular ejection fraction of 50%. ECG revealed normal sinus rhythm, and vaginal delivery with the possible operative second stage was planned with induction at 39w0d. Anesthesiology was consulted on admission for epidural consultation. Induction was initiated with vaginal misoprostol and cervical ripening balloon, and the patient progressed to complete dilation after 10h45 min with a continuous lumbar epidural in place. Telemetry was maintained through labor and delivery and remained with normal sinus rhythm. The patient delivered a healthy 3.08 kg baby boy in a normal vaginal delivery without complications after a second stage of 1h19 min. Flecainide was discontinued on delivery and the patient was maintained on metoprolol.

## Discussion

Tachycardia-induced cardiomyopathy is a disease of the heart muscle caused by long-standing tachycardia, which in most instances is treatable with a good prognosis and can occur in all types of tachyarrhythmias. These tachyarrhythmias can lead to marked depression of left ventricular ejection fraction, elevated filling pressures, depressed cardiac output, and increased systemic vascular resistance. Treatment of the underlying etiology is essential to the management and the reversible nature of cardiomyopathy. The focus in treatment is on both rate and rhythm control strategies. The clinical presentation of tachycardia-induced cardiomyopathy is often asymptomatic or can be presented with palpitations until the patient develops frank heart failure [1].

Atrial tachycardia is defined by a heart rate of more than 100 beats per minute that originates outside of the sinus node [2]. Incessant or atrial tachycardia that is present for at least 90% of the monitored time can lead to tachycardia-induced cardiomyopathy [3].

Most patients are present with palpitations, dizziness, sensation in the chest, or chest pain. If atrial tachycardia leads to tachycardia-induced cardiomyopathy, presenting symptoms could be shortness of breath, extreme lower edema, and chest pain [4].

Pregnancy is associated with an increased incidence of arrhythmia. A study of pregnant patients with cardiac arrhythmias detected by Holter monitoring showed that repeated Holter monitoring six weeks postpartum in nine women with multiple premature contractions during pregnancy (9,073 ± 9,210/24 hours) showed a substantial reduction to 1,345 ± 1,997/24 hours (p <0.05) [5]. In some cases, pregnancy can exacerbate an underlying arrhythmia. In general, there is no clear mechanism of pregnancy that can trigger or exacerbate arrhythmia, but it is likely a combination of changes in hemodynamics, hormone levels, and autonomic regulation. Volume can play a significant role as effective circulating volume increases by 30% to 50% from 8 weeks of gestation to 34 weeks of gestation. Cardiac output also increases via an increase in stroke volume of approximately 35% and an increase in heart rate of approximately 15%. A high resting rate itself can be arrhythmogenic. An increase in circulating volume causes stretch of the myocytes which can result in changes in conduction, depolarization, and activation of stretch-activated ion channels. Estrogen and progesterone are also shown to have arrhythmogenic properties. Also, estrogen increases the number of adrenergic receptors in the myocardium and adrenergic responsiveness seems to be increased in pregnancy [6].

There is no defined limit for “physiological tachycardia” in pregnancy and because of that, physicians must be able to identify at what point patients need additional evaluation for elevated heart rate. A key recommendation from the Mothers and Babies: Reducing Risk through Audits and Confidential Enquiries across the UK (MBRRACE-UK) 2019 report is the importance of investigating ‘a persistent sinus tachycardia’ as this is considered a red flag, particularly when there are associated symptoms such as breathlessness or chest pain [7]. It is important to perform an echocardiogram on these patients to evaluate for LV dysfunction. 

In general, the therapeutic approach to arrhythmias during pregnancy is similar to that of the general population and treatment should be reserved for those with significant symptoms or arrhythmias resulting in hemodynamic compromise and therefore risk to the patient and fetus. Overall, there is not enough data on the efficacy and safety of antiarrhythmic drugs in pregnancy, and most are categorized as class C (risk cannot be ruled out) in the US.

Digoxin, calcium channel blockers, and β-blockers can be used for rate control in pregnant patients. If those agents fail to provide adequate control, flecainide or sotalol should be considered. Catheter ablation should be considered if the patient is refractory to medical therapy and if potential risks such as fetal radiation exposure and fetal compromise in the event of maternal hemodynamic instability are outweighed by the benefits [8].

In addition, we would like to point out that COVID-19 also is a well-known cause of cardiac arrhythmia as well as other cardiac complications including heart failure and acute coronary syndrome however this is not a topic of this case report. [9]. In vivo studies on mice and rabbits infected with SARS-CoV demonstrated direct viral RNA inclusion in cardiomyocytes and conduction system disease however more studies need to be performed to determine true arrhythmogenic properties of COVID-19, especially during pregnancy [10].

## Conclusions

Tachycardia during pregnancy is often interpreted as a normal finding associated with a physiological heart rate increase. However, as discussed above there is no clear cut-off value when tachycardia is pathological during pregnancy. It is very easy to overlook the potential development of tachycardia-induced cardiomyopathy because symptoms can also be masked under physiological pregnancy symptoms. In this case report, we wanted to bring the attention of the providers on every level to pay closer attention to tachycardia during the pregnancy. An electrocardiogram is not an expensive and reliable tool to evaluate for underlying arrhythmias that can cause tachycardia. In case of persistent tachycardia and developing or worsening symptoms of shortness of breath, we suggest obtaining an echocardiogram for evaluation of left ventricular function. The authors want to point out that there is no clear-cut heart rate for “persistent tachycardia” during pregnancy and it is unclear if such a tachycardia can fit the criteria of inappropriate tachycardia. Management of tachycardia in pregnancy is challenging, due to the limitations imposed by transplacental drug transfer and the physiologic increased volume of distribution in pregnancy. Though labetalol is one of the agents of choice for hypertensive disorders of pregnancy, it has alpha-blocking activity and intrinsic sympathomimetic activity, whereas metoprolol is cardio-selective. In a pregnant patient who was borderline hypotensive at baseline, we selected metoprolol for its favorable effect on heart rate without alpha blockade. In addition, flecainide is easier to dose and requires less monitoring than digoxin, which has a narrow therapeutic window. Sotalol would be another antiarrhythmic option that was not deemed necessary in this case as good heart rate control was achieved with metoprolol and flecainide. There is limited data on the safety of antiarrhythmics during pregnancy; however, pregnant people who suffer from significant tachycardia, especially when complicated by tachycardia-induced cardiomyopathy should be treated with a beta blocker, digoxin and, if the heart rate is not controlled, an antiarrhythmic like flecainide or sotalol.
